# Percutaneous Cementoplasty to Treat Sternal Instability After Cardiac Surgery

**DOI:** 10.3389/fcvm.2022.822540

**Published:** 2022-02-07

**Authors:** Thaïs Pittet, Stéphane Cook, Gregory Khatchatourov, Nicolas Theumann

**Affiliations:** ^1^Cardiology Department, University Fribourg, Fribourg, Switzerland; ^2^Hirslanden Clinics Lausanne, Lausanne, Switzerland

**Keywords:** sternotomy, sternal non-union, percutaneous cementoplasty, heart surgery complications, coronary artery bypass graft (CABG)

## Abstract

**Introduction:**

Although rare, sternal pseudarthrosis is encountered after cardiac surgery and impacts the quality of life by triggering motion-dependent chest pain. We thought to describe its treatment by percutaneous cementoplasty and report the clinical follow-up of patients treated in our institution.

**Methods:**

This case series is a retrospective study based on five patients who benefited from a sternal cementoplasty as a treatment for symptomatic pseudarthrosis after cardiac surgery. The progression of the symptoms was assessed during clinical follow-up using the Quebec back pain disability (QBPD) scale and Visual Analog Scale (VAS).

**Results:**

None of the patients presented evidence of local complications or neurological disorders. The intra- et post-operative images show no major leak of the cement, no embolism and no damage to the internal mammary artery or the heart. All patients described an improved quality of life due to reduced pain in all-day clinical activities. The QBPD scores improved from 54.8 ± 29.3 to 30.0 ± 17.4 (*p* = 0.02) and the VAS from 7.0 ± 2.8 to 1.6 ± 1.6 (*p* = 0.01). Furthermore, three out of five patients could completely stop taking analgesics.

**Conclusion:**

Sternal pseudarthrosis is a debilitating affliction that may complicate sternotomy after cardiac surgery. This series demonstrates that a more conservative approach such as cementoplasty can be successful in terms of reducing pain, and constitutes a promising technique in selected cases.

## Introduction

Median sternotomy is the most common approach for cardiac surgery. This technique is considered safe but pertains a complication rate of 0.5 to 5% (1–3). Sternal instability is the most frequent complication encountered. During the first 2 weeks after surgery, sternal instability is named dehiscence. After 3 months, it is named pseudarthosis (4). Sternal pseudarthrosis (SPA) is defined as a defect of consolidation with no sign of healing and is characterized by sternal instability. This is characterized by sternal clicking, excessive motion discomfort (4), and pain in the absence of infection (5). The incomplete sternal union might be due to scarce sternal stability because of intensified thoracic motions, an exaggerated fibrous tissue covering the osteotomy line, and/or an impaired healing capacity. Patient (diabetes mellitus, obesity, chronic obstructive pulmonary disease, smoking) and procedural (cardiopulmonary bypass, excessive use of bone wax, bilateral internal mammary artery harvesting) risk factors have been considered (1, 4, 6–9). Sternal pseudarthrosis (SPA) significantly impacts the quality of life of suffering patients and increases their risk of pneumonia and mediastinitis (2, 4, 10).

The pain found in SPA is due to the friction of the bone with fibrous tissue. The cement acts in two ways: it reduces the mechanical stress and heats up to 70°C during its polymerisation leading to the destruction of the nerve endings. It already reaches 70% of its solid-state within 20–25 min after injection and is completely compact after 4 h. In SPA, the cement decreases micro-movements and stimulates subsequent bone consolidation. The cement's bridges have good resistance to axial forces, and the remaining sternal wires ensure vertical stability.

We describe hereby the characteristics of five patients suffering from SPA and who underwent percutaneous cementoplasty in our center.

## Patients and Methods

All patients treated for SPA in 2017 were included. A *sternal instability scale* (SIS) was used to assess SPA (11). The assessor evaluates the instability by placing two fingers on each side of the incision while the patient performs unilateral and bilateral shoulder flexion and abduction, trunk rotation and lateral flexion, cough and profound inspiration. Indeed, upper limbs' mobilization causes longitudinal shear, whereas cough and deep inspiration put the sternum through lateral and transverse shear (8, 12). Those three distraction forces, particularly lateral shear, are responsible for sternal split (2). The SIS grading ranges from 0 to 4, ranging from no detectable motion to complete instability with up to >1 ½ finger space (11).

The baseline clinical and procedural characteristics were obtained by reviewing the hospital electronic database. Once SPA was considered, its characterization was performed by computed tomography (CT) imaging to delineate the extent of the malunion and the presence or absence of bone bridges.

### Cementoplasty Technique

After performing local anesthesia with lidocaine, the procedure is executed under sedation-analgesia with morphium, midazolam and intravenous paracetamol. Antibiotic prophylaxis is made with 1.5 g intravenous cefuroxime. The intervention is achieved under CT-scan imaging to verify the correct placement of the needles and the spreading of cement. All CT-guided injections are performed with one 64-channel multidetector CT scanner (Somatom Definition AS; Siemens Healthineers, Erlangen, Germany). A monoplanar CT scout view was obtained in the lateral projection (tube current: 60 mAs, peak voltage: 120 kVp), followed by a spiral CT scan (tube current: 50 mAs, peak voltage: 100 kVp), as preparation for the injection. The pre-injection CT images is limited to the sternal segments of interest to minimize the radiation exposure. The needle puncture site on the skin is selected on the initial spiral CT scan sections.

Spinal Confidence™ combines a highly viscous cement with a hydraulic distribution system. The ultra-viscous radiopaque cement CONFIDENCE™ (DePuy Synthes, Johnson&Johnson) reaches a pasty phase immediately after the cement components have been mixed, thus limiting the risk of intravascular embolization. The amount of cement spread is let at the discretion of the in-charge radiologist. Bedrest is then taken for 4 h to allow the cement to consolidate.

### Follow-Up

As the intervention is made under CT-scan and the cement is almost stiff when the procedure ends, there is no need for a radiological follow-up unless the patient presents new symptoms. One month after the cementoplasty, a clinical appointment is scheduled during which the patient fills the QBPD scale and VAS and some exercises from the sternal instability scale are performed.

## Results

### Patient Demographics

Three women and two men (age 72 ± 4yo; range: 67–77) suffering from non-infected SPA were treated by percutaneous cementoplasty (Table 1). Three of them had a metabolic syndrome with obesity (BMI >30 kg/m^2^), type-2 diabetes mellitus and dyslipidaemia. One patient suffered from chronic obstructive pulmonary disease (COPD). Four patients were smokers (64 ± 25 pack-years; range: 45–100). All patients underwent coronary artery bypass surgery with left internal mammary artery grafting and three had bilateral internal mammary artery grafting. Two patients suffered from sternal infection after CABG surgery and one patient had a redo operation. SPA was localized in three patients (manubrium *n* = 1; body *n* = 2) and generalized in two.

**Table 1:** Patient's characteristics.

**Patients**	**Gender**	**Age (years)**	**Time from operation to cementoplasty (months)**	**Obesity (> 30 kg/m^2^)**	**Diabetes**	**COPD**	**Smoking (pack-years)**
#1	M	77	11	–	–	–	Never
#2	F	74	33	–	–	–	Former, 60 p-y
#3	M	67	60	+	+	–	Former, 50 p-y
#4	F	69	30	+	+	+	Former, 45 p-y
#5	F	78	23	+	+	–	Current, 100 p-y

*+, present; −, absent*.

### Cementoplasty Procedures

**Figure 1:**
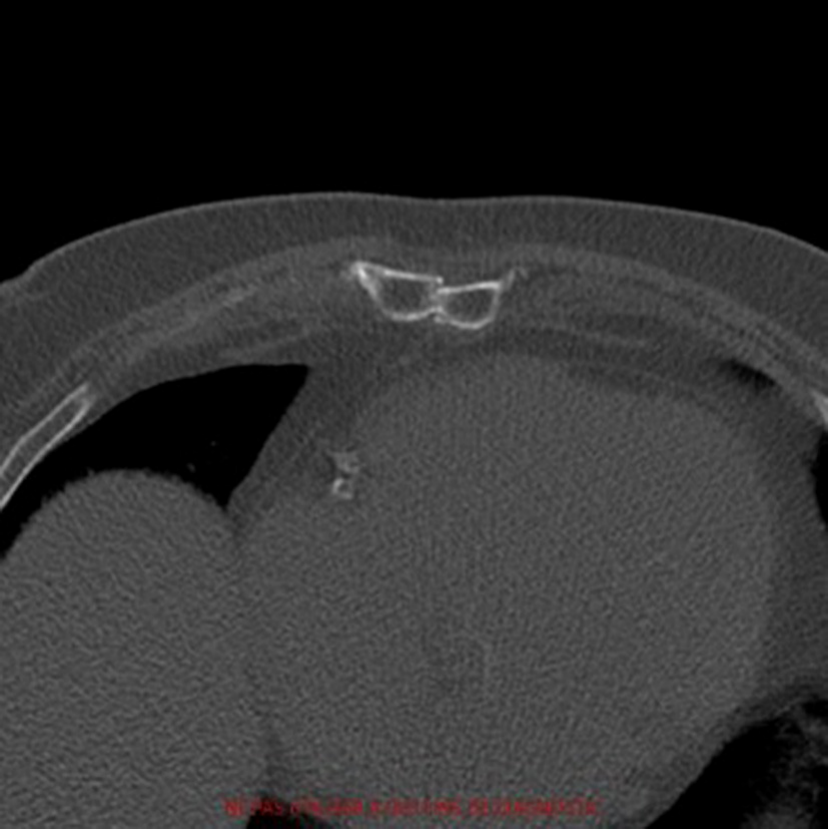
Pre-operative axial computed-tomography image of patient 1. The diagnosis of pseudarthrosis is clear as we can't see any bone bridges between the two halves.

**Figure 2:**
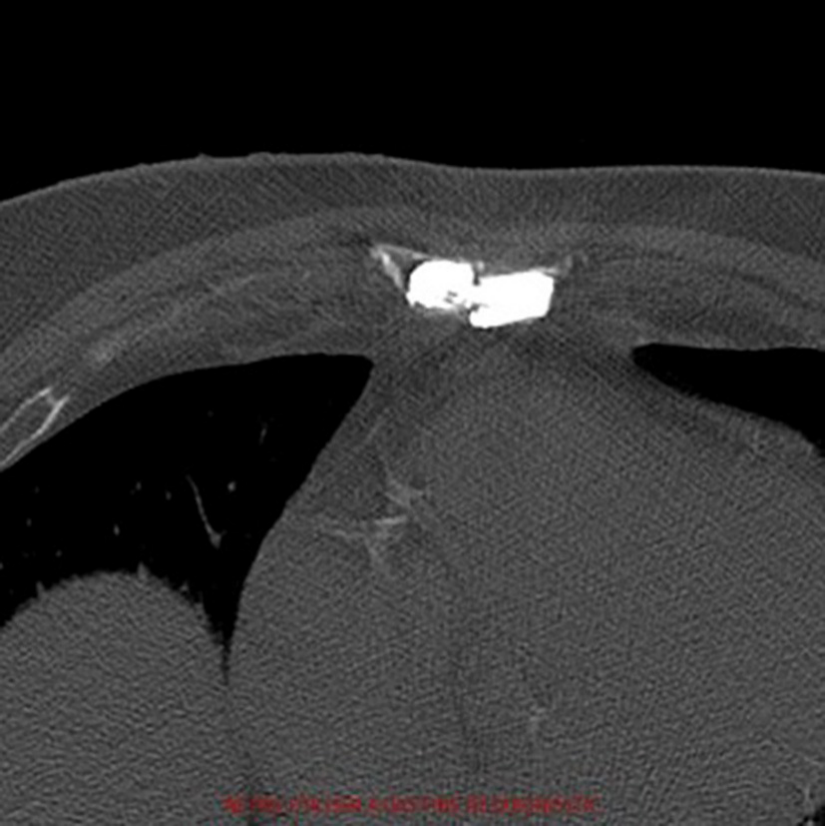
Post-operative axial computed-tomography image of patient 1. The radiopaque cement fills the pseudathrosic parts of the sternum to reduce the pain and a bridge is created for a better stabilization.

**Figure 3:**
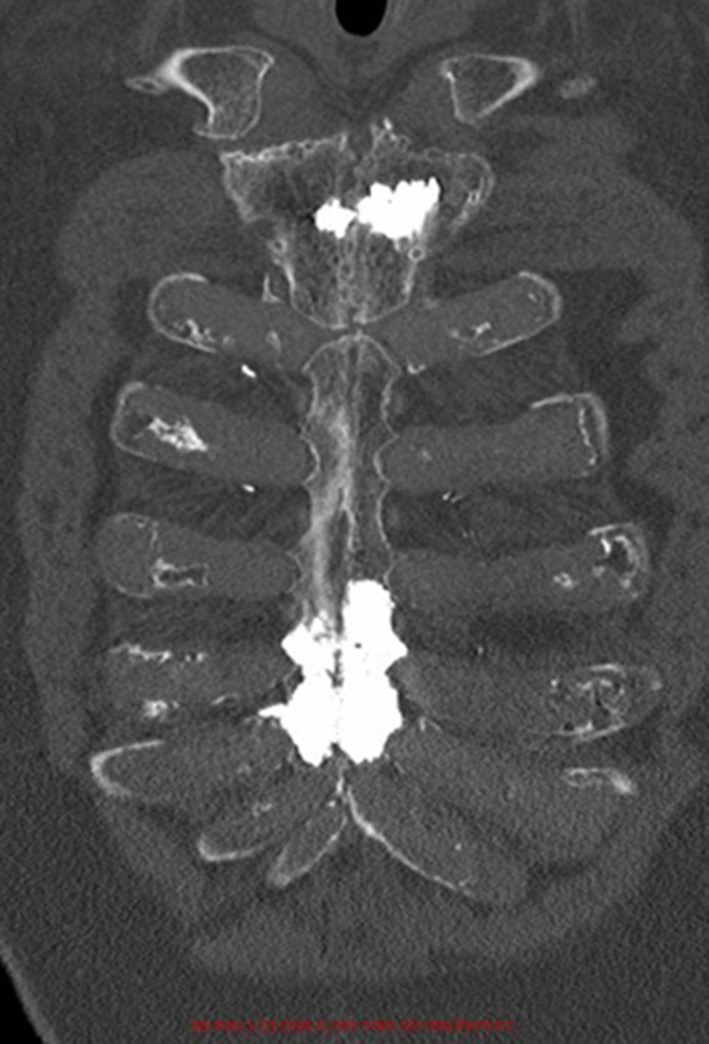
Post-operative coronal computed-tomography image of patient 1.

**Figure 4:**
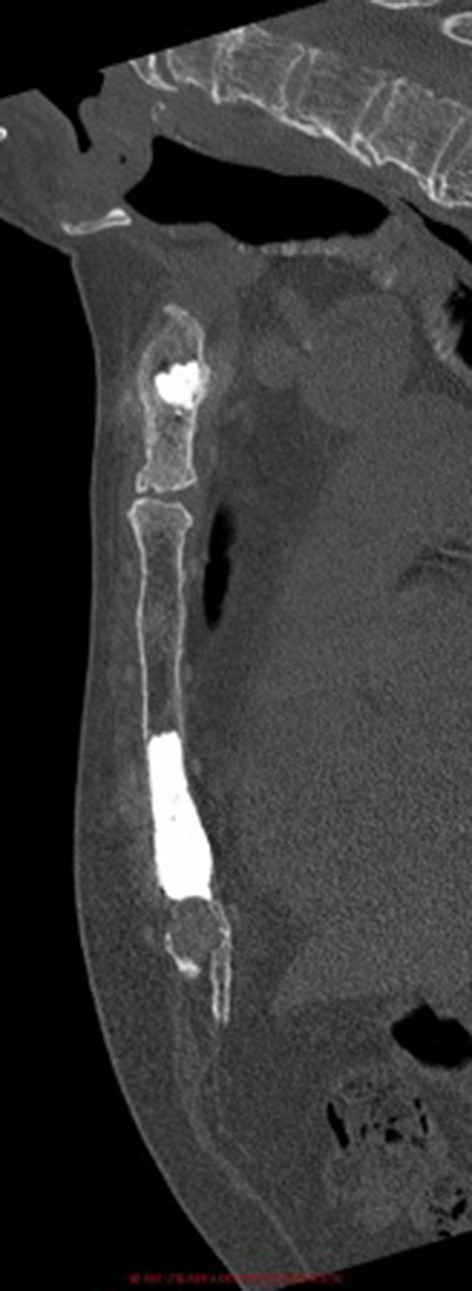
Post-operative sagittal computed-tomography image of patient 1.

The procedure time was 27 ± 12 minutes. The radiation exposure was 0.13 ± 0.04 mGy. During the procedure, 3 ± 3 mL of cement were injected. Figures 1–4 depict example of pre- and postinterventional CT findings.

No immediate or short-term complication happened. The intra- and post-operative images show no major leak of the cement, no embolism and no damage to the internal mammary artery or the heart.

#### Clinical Follow-Up

At one-month follow-up, no patients presented evidence of complications or neurological sensorimotor disorder. Every patient described an improvement in their daily activities and quality of life. The Quebec back pain disability scale score (Table 2) improved from 54.8 ± 29.3 to 30.0 ± 17.4 (*p* = 0.03) and concerning the visual analog pain scale, we noted an improvement from 7.0 ± 2.8 to 1.6 ± 1.6 (*p* = 0.01). Furthermore, three out of five patients completely stopped taking painkillers whereas two additional significantly decreased the dose.

**Table 2:** Patients' scores from the Quebec back pain disability scale before and after the intervention.

**Patients**	**QBPD scores before cementoplasty**	**QBPD scores after cementoplasty**	**Sites of injection (Manubrium (M) and/or body (B))**
1	64	46	M + B
2	5	1	B
3	56	33	M
4	68	40	B
5	81	30	M + B

## Discussion

Percutaneous cementoplasty was developed for vertebroplasty in 1984 as a treatment for vertebral hemangioma. Its indication has been extended to vertebral fracture due to its capability to tolerate compressive pressures. Here, we report for the first time the possibility of using percutaneous cementoplasty to treat SPA. We demonstrate the feasibility, the safety and the efficacy of this novel and minimally invasive alternative technique to surgery. We are convinced that percutaneous cementoplasty can be used in all type of SPA regardless of its cause.

Postoperative sternal wound infection is found in 0.5–8.4% of cases (13) and could lead to SPA. The classical risk factors for SPA are smoking, metabolic syndrome and COPD (6). In addition to increasing the odds of sternal dehiscence by 3.9 times, obesity seems to be the most critical risk factor for postoperative sternal infection (9). Moreover, Seyfer et al. noted that bilateral mammary artery grafting is at greater risk since every mammary artery harvesting is associated with a 90% drop of the ipsilateral sternal blood flow (14). Therefore, smoking, metabolic syndrome, obesity, COPD and bilateral mammary artery grafting are associated with SPA (1, 15). To limit the incidence of SPA, the modifiable components of these risk factors should be tightly controlled, and the use of steroids and non-steroidal anti-inflammatory drugs minimized during the few weeks before CABG (5). Considering that the separation of the sternal edges usually begins in the caudal third due to the concentration of distraction forces and low blood supply, adding wires at the lower end is an efficient way to lower its incidence (2, 4, 9).

When SPA occurs, a careful physical examination is mandatory during the first year since a large part of non-infectious SPA will resolve spontaneously over time. If not, a CT scan of the sternum should be done. In these patients, surgical closure or percutaneous cementoplasty will be discussed on a case-by-case basis with the in-charge surgeon. In the case of a poor surgical candidate, percutaneous cementoplasty appears to be associated with encouraging preliminary results.

### Limitations

The present study is limited in size with possible selection bias. Given the uncertainty around point estimations, extrapolations should be drawn with caution. The fact that it was performed in a single center with cementoplasty performed by a single experienced operator with uniform procedural strategies makes generalizations to other centers limited.

## Conclusion

SPA is a debilitating affliction that may complicate sternotomy after cardiac surgery. Percutaneous cementoplasty constitutes a promising technique in selected cases.

## Data Availability Statement

The original contributions presented in the study are included in the article/supplementary material, further inquiries can be directed to the corresponding authors.

## Ethics Statement

The studies involving human participants were reviewed and approved by La Commission cantonale d'éthique de la recherche sur l'être humain (CER-VD). The patients/participants provided their written informed consent to participate in this study. All patients gave their written informed consent to the procedure and the work has been performed under the general approval by the Local Ethics Committee (FR-339-14).

## Author Contributions

TP: collected the informations in patients' files, interviewed NT, and wrote the article. SC: patients' cardiologist, direct supervisor of TP, and corrected the temporary versions of the article. GK: performed the CABG and corrected the final version of the article. NT: performed the percutaneous cementoplasty and corrected the final version of the article. All authors contributed to the article and approved the submitted version.

## Funding

The trial was supported by an unrestricted grant from the Fonds Scientifique Cardiovasculaire (Fribourg, Switzerland).

## Conflict of Interest

The authors declare that the research was conducted in the absence of any commercial or financial relationships that could be construed as a potential conflict of interest.

## Publisher's Note

All claims expressed in this article are solely those of the authors and do not necessarily represent those of their affiliated organizations, or those of the publisher, the editors and the reviewers. Any product that may be evaluated in this article, or claim that may be made by its manufacturer, is not guaranteed or endorsed by the publisher.
